# Complete annotated genome sequence of *Microbacterium foliorum* phage Delphidian, isolated from soil in Philadelphia, Pennsylvania

**DOI:** 10.17912/micropub.biology.001490

**Published:** 2025-04-18

**Authors:** Dondra S. Bailey, Alison Moyer, Muyang Chunga, Prajanya Prabakaran, Bhumika Parnerkar, Dominique Dotson, Mariam Allison, Shardaye Beasley, Annalyse Belton, Candace Braxton, Delonn Dixon, Taylor Fullwood, Monique Hines, Tremaine Holmes, Tochi Iwuji, Mysia Johnson, Braxton Kess, Atiatunur Kukoyi, Erin Laster, Tanae Moore-Buchannon, Khalil Oliver, Kyara Parham, Seetra Parris, Alysha Pulliam-Robinson, Lashawna Robinson, Marcus Smith, Christiana Whitfield, Kayla Whitfield, Viviana Wamiru

**Affiliations:** 1 Department of Natural Sciences, Coppin State University, Baltimore, Maryland, USA; 2 Biology Department, Drexel University, Philadelphia, Pennsylvania, USA

## Abstract

The bacteriophage Delphidian contains a 41,595 bp DNA genome with 62 predicted protein-coding genes and no tRNA genes. Delphidian infects *Microbacterium foliorum* NRRL B-24224 and is predicted to be lytic. Based on gene content similarity to sequenced actinobacteriophages, it has been assigned to cluster EA1.

**Figure 1. Plaque morphology of Delphidian:**
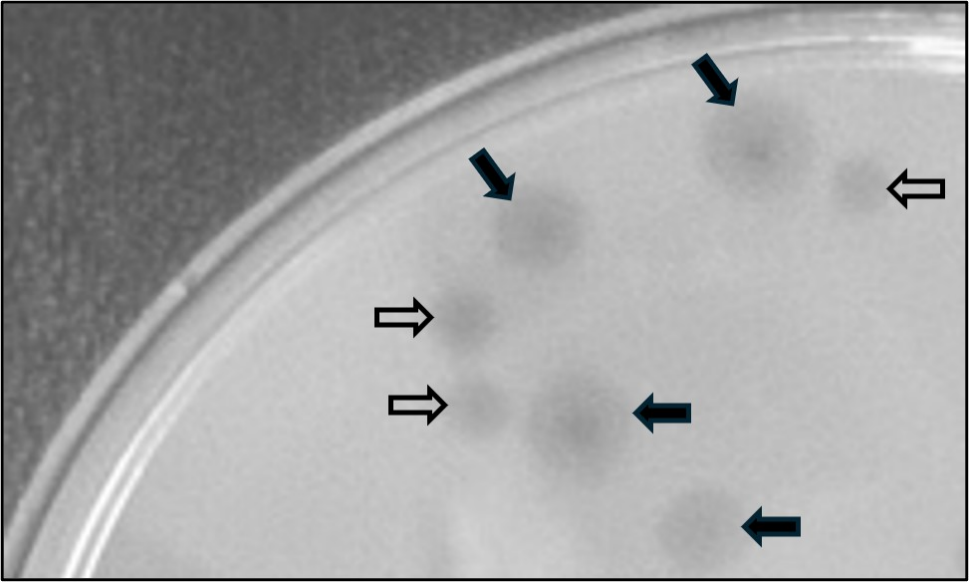
Plaques formed on *M. foliorum* on PYCa top agar overlays have clear centers and turbid halos. Plaque size varies from ~ 0.3 cm (clear arrows) to ~ 1 cm (black arrows) after incubation at 28˚C for 24 hours.

## Description

Bacteriophages, or phages, are enormously abundant, genetically diverse, and largely under-sampled. The discovery and characterization of novel bacteriophages continue to contribute to advances in biotechnology and medicine (Hatfull, 2022). Here, we describe the isolation and genetic characteristics of the bacteriophage Delphidian, isolated using *Microbacterium foliorum* ( *M. foliorum* ) NRRL B-24224 (Russell et al. 2019).

Bacteriophage Delphidian was isolated from a damp soil sample collected on the campus of Drexel University in Philadelphia, Pennsylvania (global positioning 39.95435 N, 75.18837 W) using standard methods (Zorawik et al., 2024). The resulting culture was filtered (0.2 µm pore size) and the filtrate plated with *M. foliorum* PYCa top agar overlays, resulting in visible plaques after incubation at 24 hours at liquid medium for 2 hours at 28°C and, after centrifugation (2,000 x g), filtered through a 0.2 µm filter. The filtrate was inoculated with a late log-phase *M. foliorum* culture and incubated with shaking for 24 hours at 28 ˚C. Bacteriophage Delphidian was plaque-purified via three rounds of plating and produced plaques of variable size with a clear center and turbid halos (Figure 1).

Genomic DNA was extracted from a phage lysate using the Promega Wizard DNA Kit. A sequencing library was prepared using the NEB Ultra II Library Kit and sequenced using an Illumina MiSeq (v3 reagents), which resulted in 526,005 single-end 150 base reads and 1,076x coverage. Raw reads were assembled using Newbler v2.9 and checked for completeness using Consed v29, using established protocols (Gordon et al., 1998; Russell 2018). Delphidian genome is 41,595 bp long, circularly permutated, and has a GC content of 63.5%.

The genome was automatically annotated using DNA Master (cobamide2.bio.pitt.edu; v5.23.6, build 2705 24 October 2021), embedded with Glimmer (v3.02b) (Delcher et al., 2007) and GeneMark v2.5 (Besemer and Borodovsky 2005). Start sites were refined utilizing Starterator v485 (http://phages.wustl.edu/starterator/) and Phamerator, the latter using the Actino_draft database v578 (Cresawn et al., 2011). Putative functions for 62 identified genes were assigned based on BLASTp v2.13.0 searches against the Actinobacteriophage and NCBI_Conserved_Domains (Altschul et al., 1990) and HHPRED searches against the PDB_mmCIF70, Pfam- v.36, and NCBI Conserved Domains databases (Söding et al., 2005). There is no tRNA present in the Delphidian genome, as assessed using ARAGORN (v1.2.41) (Laslett and Canback 2004) and tRNAscan-SE (v2.0) (Lowe and Eddy 1997). All software were used with default parameters.

Delphidian is assigned to actinobacteriophage cluster EA, subcluster EA1, based on gene content similarity of 35% or more to phages in the Actinobacteriophage database (http://phagesdb.org)(Pope et al., 2017). Delphidian adds to a growing list of over a hundred bacteriophages in subcluster EA1, and shares 99% nucleotide identity and 96.01% gene content similarity with SoilGremlin (https://phagesdb.org/phages/SoilGremlin/), another cluster EA1 phage that was isolated from a soil sample collected within 400 feet of the collection site for Delphidian. As with other EA1 phages, or cluster EA phages more broadly, Delphidian lacks an identifiable repressor gene, an integrase, or genes with DNA partitioning function and is thus unlikely to establish lysogeny (Jacobs-Sera et al., 2020).

**Nucleotide sequence accession numbers**

Delphidian is available at GenBank with Accession No. PQ362667 and Sequence Read Archive (SRA) No. SRX26311142 .
